# Deficit in visual temporal integration in autism spectrum disorders

**DOI:** 10.1098/rspb.2009.1713

**Published:** 2009-12-02

**Authors:** Tamami Nakano, Haruhisa Ota, Nobumasa Kato, Shigeru Kitazawa

**Affiliations:** 1 Department of Physiology, Juntendo University School of Medicine, Tokyo, Japan; 2 Core Research for Evolutional Science and Technology (CREST), Japan Science and Technology Agency, Saitama, Japan; 3 Japan Society for the Promotion of Science, Tokyo, Japan; 4 Department of Psychiatry, Showa University School of Medicine, Tokyo, Japan

**Keywords:** autism, slit viewing, weak central coherence

## Abstract

Individuals with autism spectrum disorders (ASD) are superior in processing local features. Frith and Happe conceptualize this cognitive bias as ‘weak central coherence’, implying that a local enhancement derives from a weakness in integrating local elements into a coherent whole. The suggested deficit has been challenged, however, because individuals with ASD were not found to be inferior to normal controls in holistic perception. In these opposing studies, however, subjects were encouraged to ignore local features and attend to the whole. Therefore, no one has directly tested whether individuals with ASD are able to integrate local elements over time into a whole image. Here, we report a weakness of individuals with ASD in naming familiar objects moved behind a narrow slit, which was worsened by the absence of local salient features. The results indicate that individuals with ASD have a clear deficit in integrating local visual information over time into a global whole, providing direct evidence for the weak central coherence hypothesis.

## 1. Introduction

Autism spectrum disorders (ASD) are characterized by impairments in social cognition and communication. Meanwhile, segments of ability remain, typically, in processing local features (Kanner 1943). Frith and Happe conceptualize this detail-focused cognitive style as weak central coherence, implying that an enhancement in local processing derives from a weakness in integrating local elements into a coherent whole (Frith 1989; Frith & Happe 1994; Happe & Frith 2006). The suggested deficit in central processing in ASD has been challenged (Plaisted *et al*. 1999; Caron *et al*. 2006; Mottron *et al*. 2006), however, because individuals with ASD are not found to be inferior to normal controls in holistic perception, at least when it is required of them. Results have been inconsistent, even when tasks were designed to make attention to a local feature compete with attention to a global feature, as in Navon hierarchical figures (e.g. an H composed of small Ss; Plaisted *et al*. 1999). These reports raise a critical question as to whether the theory of weak central coherence is valid at all.

In these previous studies, however, subjects were not required to combine local features to construct a global image of a whole. Rather, they were encouraged to ‘ignore’ local features and attend to the whole. Jolliffe & Baron-Cohen (2001), on the other hand, examined the ability to integrate fragments of an object and found that adults with ASD were significantly impaired in their ability to integrate pieces holistically. However, pieces were drawn on a card and presented at once to the subjects in their study (Jolliffe & Baron-Cohen 2001). This leaves a possibility that the impairment in adults with ASD resulted from their attentional bias towards local features.

**Figure 1.**
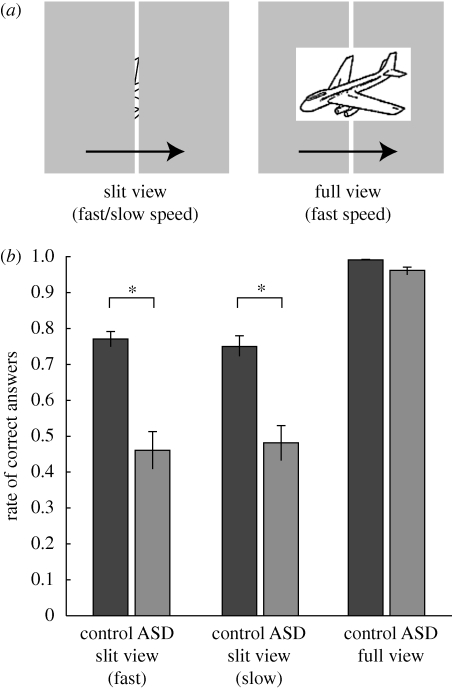
Poor performance at slit viewing in adults with ASD. (*a*) Visual stimuli in slit and full viewing conditions. A black line drawing of a familiar object (Snodgrass & Vanderwart 1980; *n* = 40) moved behind a narrow vertical slit (width: 0.14°) once for each trial, from left to right. Each object subtended approximately 3–4°. Fully visible pictures of the same objects were presented in front of the slit in the full viewing condition at the fast speed. (*b*) Performance in the ASD and control groups in the slit and full viewing conditions. Two-way analysis of variance detected significant main effects of group (*F*_1,31_ = 25.5, *p* < 0.0001) and condition (*F*_2_ = 119.0, *p* < 0.0001), and significant interactions (*F*_2,62_ = 16.2, *p* < 0.0001). Asterisks indicate significant simple main effects of the group in the slit viewing conditions (fast: *F*_1,93_ = 38.4, *p* < 0.0001; slow: *F*_1,93_ = 27.3, *p* < 0.0001).

To directly examine the ability to integrate elements into a whole image without the confounding factor of attentional bias, we asked adults with ASD and normal adults to name a familiar object moving behind a narrow vertical slit (figure 1*a*). In this task, only a single local piece of information was provided at a time through a narrow slit on which subjects focused their attention (Parks 1965). Each subject named 40 figures three times, once for each of the three blocks: in the first and the second block, pictures were presented behind the slit (slit viewing) at a fast and a slow speed, respectively; in the third block whole pictures were presented in front of the slit at the fast speed (full viewing).

## 2. Material and Methods

### (a) Subjects

This study involved 17 adults with ASD (age: 32.4 ± 8.2 years, range 21–48 years) and 16 normal adults (age: 29.4 ± 6.9 years, range 21–47 years; see table S1, electronic supplementary material). Adults with ASD met the *Diagnostic and statistical manual of mental disorders*, fourth edition (DSM-IV) criteria (American Psychiatric Association, 1994). ASD diagnoses were established based on the clinical judgement of two medical specialists. All subjects had normal or corrected-to-normal vision. Among the ASD subjects, 5 of 17 were medicated (four were taking antidepressant drugs, two were taking tranquilizers, two were taking antipsychotic drugs and one was taking an antiepileptic drug). The correct response rate in slit viewing, however, was not significantly different between medicated and non-medicated ASD subjects (two-sample *t*-test, *p* > 0.05). Informed written consent was obtained before the experiments were performed. The experimental protocol was approved by the institutional review committee.

### (b) Apparatus

The stimulus was generated using MATLAB Psychophysics toolbox on a PC (Dell T3400). It was presented on a CRT monitor (19 inch, Eizo) with a refresh rate of 100 Hz and a spatial resolution of 1024 × 768 pixels. The entire display subtended approximately 35.7 × 27.2° of visual angle. Each pixel subtended 2.1 inch at the viewing distance of 60 cm.

### (c) Stimuli

We used 40 black line drawings of ordinary objects (Snodgrass & Vanderwart 1980) that subtended approximately 3–4° of visual angle when fully visible. The aperture consisted of a simulated grey occluding surface with a white strip in the centre acting as the slit. The slit width was 0.14° (4 pixel), and the object translated behind the occlusion at 3.5° s^−1^ (100 pixel s^−1^) and 1.8° s^−1^ (50 pixel s^−1^) in the fast and slow conditions, respectively, with only a narrow slice visible at any time through the slit. The fixation point, a small white cross, was placed to the left of the slit. In the full view condition, the object moved at 3.5° s^−1^ in front of the occlusion. The object always moved from left to right once per trial.

### (d) Procedures

Each experimental session consisted of three blocks: the first two blocks were the slit viewing task (the order of the fast and slow speed conditions was counterbalanced across subjects), and the third block was the full viewing task. Subjects fixated upon the white cross during the task. In each block, 40 line drawings were randomly presented. After each presentation, subjects gave the object name, and an experimenter wrote it down. The voice of each subject was recorded by an IC recorder for later confirmation.

### (e) Data analysis

To quantify the availability of local salient features, we calculated the mean density of each picture. We counted the number of black pixels and divided that number by the area (in pixels) of the minimum rectangle that covered the whole object. Then, we examined the relation between the rate of correct response and the mean picture density by using an analysis of covariance (ANCOVA) with the rate of correct response as a dependent variable, group (ASD/normal) as a factor and mean density as a covariate.

## 3. Results and discussion

**Figure 2.**
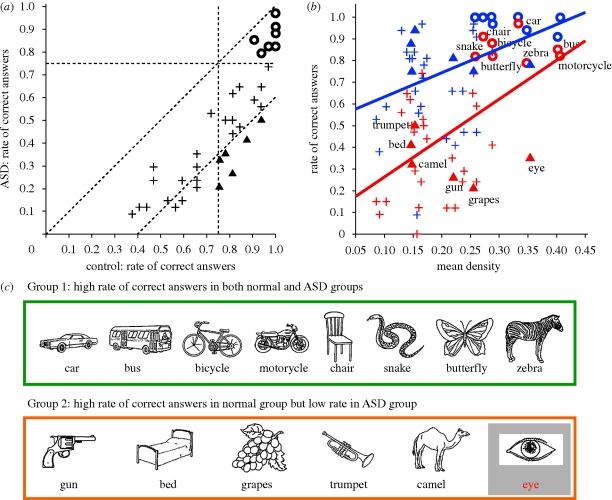
Object-by-object performance in ASD and control subjects. (*a*) Comparison of the rate of correct answers in the ASD (ordinate) and control subjects (abscissa). Circles indicate eight objects that were easily identified by both groups (greater than 75%; Group 1 figures), while triangles indicate six objects that were easily identified by the control group (greater than 75%), but difficult for the ASD group (Group 2 figures). Pluses represent the other 26 objects. Oblique lines show *y* = *x* and *y* = *x −* 0.4. (*b*) Group comparison of the rate of correct answers (ordinate) relative to the object mean density (abscissa). Red and blue represent the ASD and control groups, respectively. Shapes correspond to those in (*a*). An ANCOVA with group as a factor and mean density as a covariate showed significant effects of group (*F*_1,76_ = 10.4, *p* = 0.002) and their interaction (*F*_2,76_ = 12.5, *p* < 0.0001). Note a steeper slope of the regression line for the ASD group (red line; slope = 1.8, *t* = 4.3, *p* < 0.0001) than for the control group (blue line; slope = 1.1, *t* = 2.6, *p* = 0.01). (*c*) Group 1 and Group 2 figures.

In the slit-viewing conditions, the mean rates of correct answers in the ASD group (fast: 46%, slow: 48%) were strikingly lower than those in the control group (fast: 77%, slow: 75%) at both speeds (figure 1*b*). In addition, the rate of correct answers in ASD subjects was lower than that in the control group for every one of the 40 figures (figure 2*a*). By contrast, both groups successfully named almost all pictures in the full-viewing condition (ASD: 96%, control: 99%). Thus, the ability to identify objects was intact in ASD individuals.

Assuming that first-order motion perception is intact in ASD individuals (Bertone *et al*. 2003), their poor performance in the slit-viewing condition clearly indicates a deficit in constructing a global picture by combining local visual information presented in succession over time.

To highlight the relative strengths and weaknesses in the perception of ASD subjects, we further analysed eight figures that were easy (greater than 75% correct) for both groups to identify (circles in figure 2*a*) and six figures that were easy for the controls but not for the ASD subjects to identify (triangles in figure 2*a*; worse than control group rate by greater than 40%). By inspecting these data (figure 2*c*), we hypothesized that ASD subjects benefited from local salient features (e.g. wheels, stripes of a zebra, textures on the skin surface of a snake), which we quantified by the mean density of the pictures.

This hypothesis is supported by the finding that the rate of correct responses in ASD subjects was more highly dependent on the mean picture density (red symbols in figure 2*b*) as compared with the control group (blue symbols in figure 2*b*): both groups yielded 80 per cent correct responses at a mean density above 0.3 (circles in figure 2*b*), but a drop was more apparent in ASD subjects as the mean density decreased to 0.1. Increasing weakness in ASD subjects for a picture that lacked locally salient features indicates that the ASD subjects rely on the local features, although they were clearly instructed to identify the whole picture. Thus, the present results support the original theory of weak central coherence, which holds that the local bias in ASD subjects reflects a weakness in constructing a coherent whole.

We were successful in detecting this weakness because we did not provide the whole image of the picture at once, but presented each piece of information one-by-one over time. According to recent functional imaging studies, such integration during slit viewing involves multiple brain areas, including the ventral occipital complex and the human motion complex (Yin *et al*. 2002). In addition, the parietal association cortex might be involved in representing an object image in slit viewing given that brain-damaged patients with unilateral neglect showed contralesional neglect of constructed visual images in slit viewing (Bisiach *et al*. 1979). Therefore, temporal integration of successive visual information during slit viewing involves a distributed cortical network, including higher visual areas and parietal association areas. Thus, the long-range underconnectivity implicated in the autistic brain (Herbert *et al*. 2004; Wang *et al*. 2009) may result in a deficit in visual temporal integration across these areas.

We would like to note that, according to the data presented in figure 2*b*, ASD subjects were much worse at naming the eye (35% correct) than expected from its high density of 0.35. That the merit of local features was lost in the picture of an eye, a key target for communication, implies that a special neural mechanism for detecting eye-like stimuli (Batki *et al*. 2000; Senju *et al*. 2004) may not function normally in the autistic brain (Pelphrey *et al*. 2005; Senju *et al*. 2005). Generalization of the present finding to other ‘eye’ objects and social stimuli merits further investigation.

We have shown that individuals with ASD performed less well than normal controls when asked to name a familiar object that passed behind a narrow slit. Because subjects were forced to reconstruct the whole picture from pieces of information seen through the slit, this result clearly indicates that individuals with ASD have a deficit in integrating pieces of information received over time into the whole picture. This deficit was enhanced when the picture lacked salient local information, suggesting that they could not help but rely on the local salient cues to identify the object. This study provides direct evidence for the weak central coherence hypothesis.
